# Development of a Quality Control System Using Modal Analysis to Evaluate a Multi-Point Projection Welding Process

**DOI:** 10.3390/ma17205005

**Published:** 2024-10-13

**Authors:** Maciej Karpiński, Paweł Sokołowski, Paweł Kustroń, Zygmunt Mikno, Wojciech Jopek, Janusz Pikuła

**Affiliations:** 1 Faculty of Mechanical Engineering, Wroclaw University of Science and Technology, Lukasiewicza 5, 50-371 Wroclaw, Polandpawel.kustron@pwr.edu.pl (P.K.); 2 ASPA Sp. z.o.o., Macieja Miechowity 1, 51-162 Wroclaw, Poland; wojciech.jopek@aspa.pl; 3 Sieć Badawcza Łukasiewicz—Górnośląski Instytut Technologiczny, Karola Miarki 12–14, 44-100 Gliwice, Poland; zygmunt.mikno@git.lukasiewicz.gov.pl (Z.M.); janusz.pikula@git.lukasiewicz.gov.pl (J.P.)

**Keywords:** resistance projection welding, quality control, spectral analysis, FEM, weld quality, modal analysis

## Abstract

The article presents the results of the development and research regarding the application of modal analysis to the evaluation of a multi-point projection welding process. The quality control system is based on the information provided by modal analysis for the entire welding station after the previously completed welding process. The research is carried out due to the lack of an effective method for assessing the course of the multi-spot projection welding process in the case of a single current circuit passing through many points at the same time. A study of the applicability of modal analysis in investigating the quality of a multipoint joint was conducted for four DIN 928 nuts welded to a steel profile. The aim of the study was to determine the influence of weld defects on the displacement of resonant frequencies. To realize the objective, the dynamic properties of the entire welding station, including the sample in the frequency domain, were investigated. In the first stage of the study, a finite element method was used to perform modal analysis and examine the form of vibrations for the individual natural frequencies of the welding fixture including the sample. Then, quality verification using the dynamic resistance method was performed, which was compared later with the modal approach. The last stage of the study was to conduct modal analysis in the frequency domain to verify the numerical studies.

## 1. Introduction

The resistance projection welding technique is widely used in various industrial sectors due to its efficiency, low operating costs and the high quality of the joints created. It is particularly popular in areas such as the automotive, engineering, electrical engineering, aerospace, and white goods industries. For example, the technique is used to join nuts to components made of sheet metal of a thickness that prevents the creation of a threaded joint with adequate mechanical properties [1,2,3].

The apparatus used in the resistance projection welding process usually includes the following:

- A welding machine, which is equipped with a pressure system with prone elements. These elements allow an even distribution of the electrode pressure force to the individual welding points during the hump welding process, which contributes to the process stability [4].
- A transformer and a control system for the welding machine, which allow the adjustment of key process parameters such as welding time, the value of the pressure force, and the stabilization of the welding current [5,6].
- The welding fixture, which allows stable and reproducible positioning of the parts to be joined, while at the same time ensuring that the support points are adequately isolated from the electrical circuit and that the hump is free to settle [7,8].
- Electrodes, which supply electrical energy to the welding area, where it is transformed into thermal energy (in accordance with Joule-Lenz’s law) used to heat up the contact areas between the humps and the substrate and form a uniform joint between the parts [9].

In production practice, quality control of the completed welded joints is required. The main non-destructive quality control methods include X-ray investigations [10], modern thermal imaging methods [11], and ultrasonic testing [12,13], supported recently by extensive evaluation algorithms or machine learning [14]. These methods are used to assess the quality of the weld after the welding process; measuring dynamic resistance, for example, can be used to assess in-line quality [15]. In order to determine the dynamic resistance, it is necessary to precisely measure electrical properties during the process, such as welding current and voltage between the welding machine electrodes. Such measurements can be useful for assessing the degradation of welding electrodes [16], mainly in determining the quality of spot welds [17,18,19]. Systems based on changes in dynamic resistance have many applications [19,20]:

- Assessment and analysis of the dynamics of resistance projection welding.
- Detection of weld burnout instances.
- Identification of weld defects resulting from workpiece misalignment, insufficient clamping force, or surface contamination.
- Prediction of the weld nugget size and its mechanical strength.
- Electrode wear detection.
- Parameter adjustments to improve the quality of welds.

A characteristic of the dynamic resistance welding process is that data is recorded in the form of changes over time in process parameters such as current, voltage, resistance, and electric power, which can be used for further analysis. The model is based on a description of the physical phenomenon in order to unambiguously represent the resistance and identify defective welds. Resistance welding quality control systems extend the welding time until a predetermined resistance drop is reached. In many applications, a resistance drop of 15–20% over a reasonable welding time and with sufficient clamping force is considered to qualify the weld as correct [21,22].

The industry now needs more detailed quality control systems that can more accurately classify problems arising from the welding process. Defects occurring in today’s manufacturing lead to subtle variations that are not easily detectable by simple diagnostic techniques. In addition, the use of modern materials, such as aluminum alloys and high-carbon or coated steels, introduces differences in specification compared to low-carbon steels in terms of weld nugget formation and changes in dynamic resistance. Such materials are much more difficult to weld, and the likelihood of a weld with insufficient mechanical properties increases significantly [20,23,24].

It is worth noting that the main advances in quality control relate to spot welding, while the quality control of projection welding has not been developed to the same extent. This is due to the higher costs associated with the tooling required to properly perform the resistance hump welding process. In addition, most method research focuses on measuring a single weld, which in the case of multi-point welding in a single fixture prevents effective quality control.

To date, modal analysis has been used extensively in structural wear testing and machine diagnostics to identify anomalies that manifest themselves in resonant frequency shifts resulting from component degradation. Its successful application in these aspects leads to the conclusion of the validity of its implementation in manufacturing processes for joint components that are highly sensitive, such as in the aerospace and automotive industries, where the proper connection of components and verification of the joint are essential [25,26].

The aim of the research is to investigate the possibility of using spectral measurements to determine the natural frequency of the welding system. Such a measurement would make it possible to control the quality of workpieces made by multi-point hump welding in a single fixture.

## 2. Materials and Methods

### 2.1. Details and Test Bench

**Figure 1:**
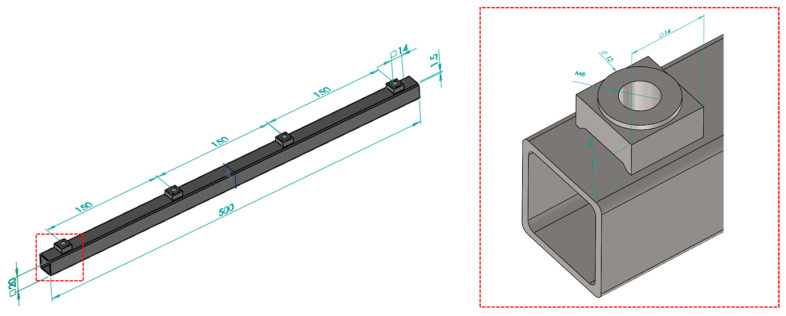
Square profile with four M8 DIN928 nuts from S235JR steel.

The specimen for this research was selected in the form of a 20 × 20 square profile with a length of 500 mm and a wall thickness of 1.5 mm (Figure 1). Four M8 DIN928 nuts were welded symmetrically to the profile with a 150 mm interval. The welding parameters are shown in Table 1.

**Figure 2:**
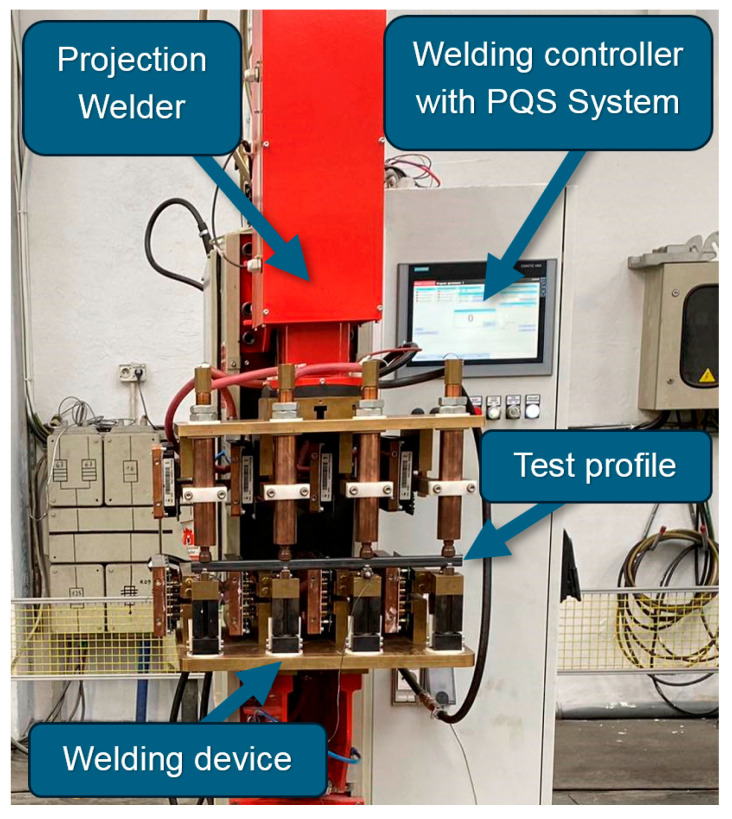
ASPA projection welder ZGm-350i equipped with welding fixtures, a PC with a welding controller, and PQS System.

The welding set-up (Figure 2) consists of the following components:

- The ZGm-350i projection welder, manufactured by ASPA (Wroclaw, Poland), is equipped with a transformer, inverter, and control system from Harms & Wende (Hamburg, Germany). The clamping system of this welder is capable of generating a maximum clamping force of F=19 kN, and the welding system can generate a maximum short-circuit current of $I_{2max}=49\;kA$.
- Welding fixtures for the projection welding of a square profile with nuts. The welding fixture allows clamping in an unambiguous and reproducible manner and compensates for differences in workpiece manufacturing tolerances by means of spring packs located in the columns and plugs, which ensure uniform clamping force.
- A PC with welding control system, namely the PQS System.

**Figure 3:**
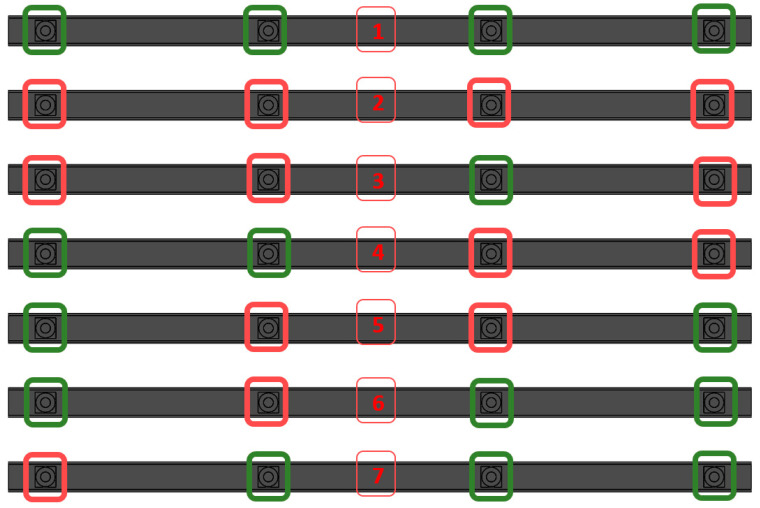
Detailed overview of samples: 1—reference detail; 2—profile with a defect in each nut; 3—profile with a defect nut 1, 2, and 4; 4—profile with a defect nut 3 and 4; 5—profile with a defect nut 2 and 3; 6—profile with a defect nut number 2; 7—profile with a defect nut number 1 (green square indicates a properly executed welded joint, a red square indicates an incorrectly made welded joint).

To carry out the tests, a series of samples were made with components with different configurations of filed humps in the nuts. The following illustration (Figure 3) shows the configurations of the welded details; green color indicates properly welded nuts, and red color indicates nuts with discrepancies. The defect in the nuts is simulated by filing down the two humps.

### 2.2. Numerical Modal Analysis (FEA)

The numerical finite element analysis (FEA) was performed utilizing the ANSYS Mechanical 2023 R1 software, a specialized tool for structural analysis employed by engineers and designers. This software facilitates the simulation of static, dynamic, thermal, and mechanical stress environments, enabling accurate predictions of product performance under real-world conditions. The aim of the study was to explore the mechanical phenomena occurring in the structure under analysis. The test object consists of a welding fixture with a workpiece fixed inside between the electrodes of the fixture. The assembly is subjected to numerical analysis, to show clear differences in natural frequency displacement between the samples. The result of the natural frequency displacement subjected to appropriate analysis will enable the quality of the welded joint to be verified. The fixture is restrained at the lower and upper overhang of the welding machine, and a force of 12 kN generated by the machine’s clamping system is applied in turn to the fixture’s top plate.

**Figure 4:**
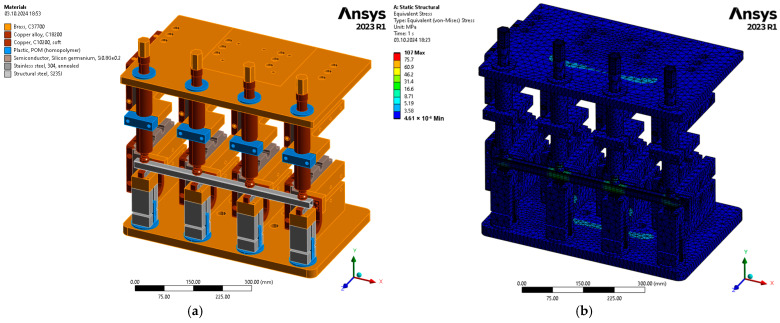
The numerical model: (**a**) materials used with the actual tooling and (**b**) results of the model static loading test.

The assumptions made for the numerical modelling were as follows:

- Material properties consistent with the actual tooling were given (Figure 4a).
- A model grid was adopted consisting of 258,501 elements and 498,119 nodes. The size of the element was 10 mm, and the grid had applied adaptive sizing and mesh defeaturing. A Jacobian ratio (Gauss points) was used for evaluating how the shape of a given element compares to the shape of an ideal element. In the numerical experiment, the Jacobian ratio was min = 0.16; max = 1; average = 0.94.
- The welding machine model was omitted; this is due to the simplification of the structure used for the simulation. It is assumed that the bottom overhang together with the top overhang form a micro-array of a rigidly restrained jig.
- A boundary condition in the form of gravity is imposed.
- The displacement force was assumed to be the clamping force of the welding machine, for which a value of 0.05 mm was determined (approximately equal to the clamping force of 12 kN).

- The springs in the electrode columns are considered as a rigid body (the actual dynamics of the springs were neglected due to their maximum deformation as a result of the machine clamping system).
- Models of fasteners were omitted due to the long computational time of the issue (the study is intended to verify the change of resonant frequencies in the absence of a mechanical connection between the profile and the nut, with the bolts in this case being an additional oscillating mass, which only displaces the point of occurrence and does not significantly affect the distinction between them for individual specimens).
- The model tested is a square with a profile of 20 mm × 20 mm × 1.5 mm, 500 mm long, with four DIN928 nuts welded on, which are made of S235JR steel (Figure 1).
- The absence of a weld between the profile and the nut is simulated by changing the contact type—between the nut and the profile—to ‘Noseparation’, while correctly welded workpieces have a ‘Bonded’ contact type.
- Contact between the top electrode and the profile and between the bottom electrode and the nut is defined as ‘Bonded’ (due to non-linearities in the analysis with a profile without a welded nut, a numerical analysis could not be performed) [20].
- Modal analysis is carried out for a frequency band coinciding with the experimental study of f∈0−5000 Hz.
- The modal test included an initial static load test condition deformation (Figure 4b).

### 2.3. Dynamic Resistance

Quality control using the method of monitoring resistance changes over time was carried out using the System PQS software v4.3.13.6738 developed by Harms and Wende.

The main functions of the PQS system include

- Taking RMS value measurements and analyzing waveforms of various signals, such as current, voltage, resistance, power, force and displacement;
- The possibility to work with any welding controller;
- Process monitoring based on predefined limit values and/or signal curves;
- The recording of process data in a database with long-term storage;
- The recording of batch or component identifiers;
- The central management of up to 16 measuring points on a single computer;
- The easy integration of the system into the Genius welding control system;
- The possibility to connect the machine via various fieldbus systems or 24 V I/O;
- Quality control through process monitoring.

In order to investigate the occurrence of incompatibility between the samples, the resistance waveforms over time during the multi-point hump welding process of the samples were analyzed. On this basis, it was analyzed whether the resistance measurement method can detect an inconsistency in the form of filed-off humps.

The software collects real-time values of voltage and current during the resistance welding process. Once the process is finished, it generates a graph showing the characteristic values over time. In addition, the software allows process data to be exported, enabling the recorded values to be analyzed in other software. This data enables further analysis of process variations to ensure the quality of the components.

### 2.4. Experimental Modal Analysis

The modal analysis is a well-known and widely used method for detecting structural failures in machine diagnostics, but so far, this method has not been used to evaluate the multi-spot projection welding process. The main emphasis in the initial research using modal analysis was placed on the selection of appropriate instrumentation and configuration, leading to obtaining a spectral response free from ambient noise, which may adversely affect further signal analysis.

**Figure 5:**
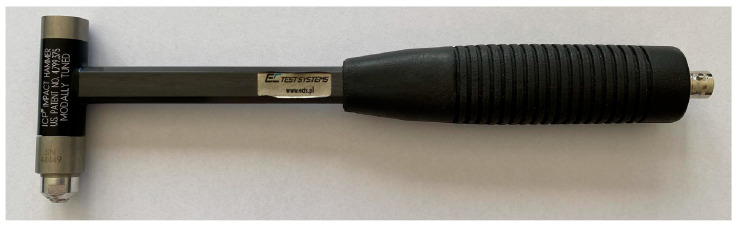
Piezotronics PCB modal hammer 086C01 from Huckelhoven, Germany.

**Figure 6:**
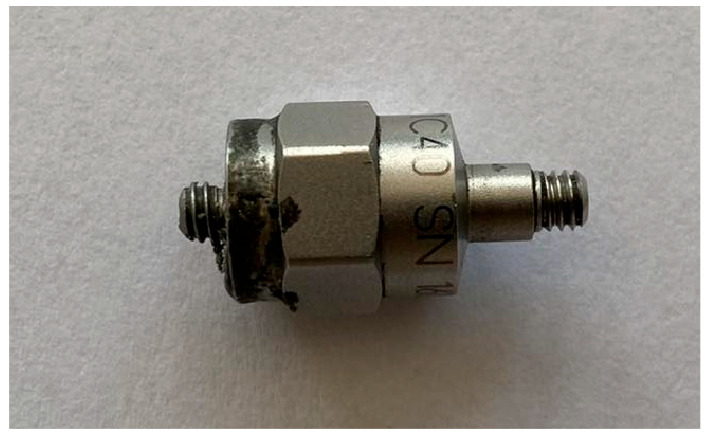
Piezotronics 621C40 PCB accelerometer.

**Figure 7:**
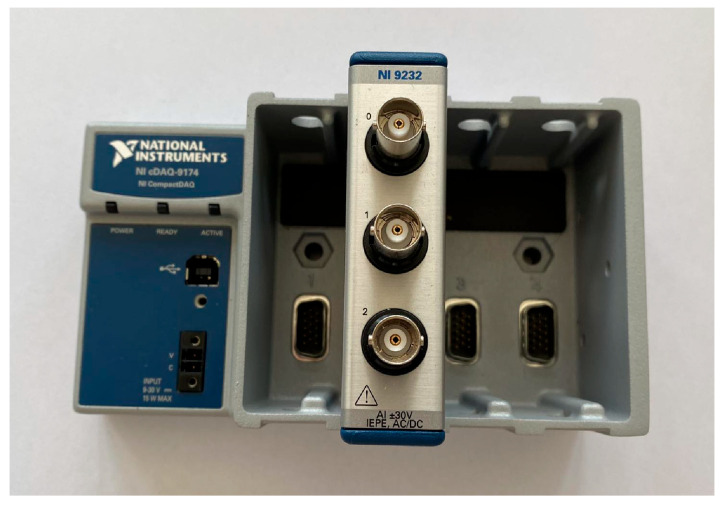
The cDAQ-9174 enclosure and NI-9232 measurement board.

The following components were selected to perform the experimental modal analysis of the workpieces in accordance with ISO 7626-5:2019 [26]:

- Modal hammer (Figure 5)—the selection of a suitable hammer was based on mechanical characteristics in the form of size and mass, which influence the values of the maximum amplitude of the response signal. Based on the mass and size of the fixture including the workpiece, a hammer model 086C01 (with operational characteristics shown in Table 2) with a maximum recorded excitation of 440 N was selected.
- Acceleration sensor (Figure 6)—the main characteristics that determine the selection of a suitable accelerometer are the mass of the sensor and the frequency range to be measured. Too great a mass of the accelerometer can generate additional signal noise, which makes further signal analysis impossible. The measuring range indicates the domain in which resonant frequency analysis will be possible. The 621C40 PCB Piezotronics accelerometer was selected (with measuring characteristics shown in Table 3).
- A National Instruments measurement and archiving card, whose purpose was to condition, activate, and convert analogue signals into discrete ones. Measurements were realized via a four-slot Compact DAQ cDAQ-9174 chassis and a 24-bit NI-9232 measurement card (Figure 7) with 3 analogue inputs. The card supports piezoelectric accelerometers in the IEPE standard, for which an additional current supply of 2–20 mA is required (measuring characteristics shown in Table 4).
- A PC equipped with signal processing software—The modal analysis application was developed in the LabVIEW 2023 Q3 23.3.2f2 environment and includes a measurement part for the acquisition of measurement data from the accelerometer and modal hammer, whose signals are analyzed in a function block designed for modal analysis. The DMQmx trigger parameter detects the signal of the excitation pulse, which initiates the recording of the response signal in a 10 s time window.

**Figure 8:**
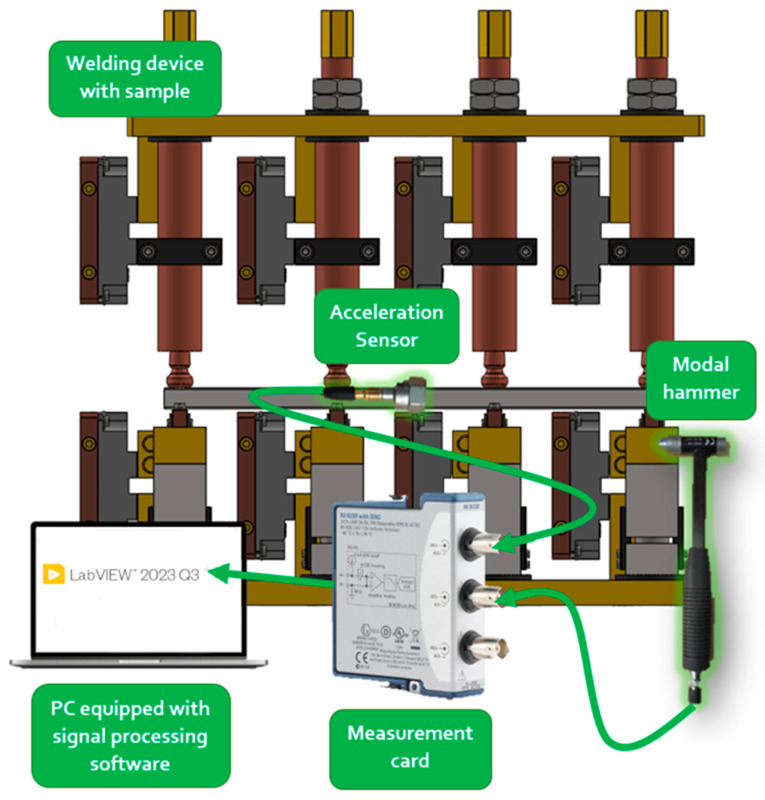
Scheme of measurement path for modal analysis.

A scheme of the experimental modal analysis carried out in this work is shown in Figure 8. The measurement path starts with an impulse excitation using a modal hammer (the force is being recorded) and observation of the object response by measuring the acceleration signal. These signals are archived via a measurement card, which transmits them to a PC via a USB interface.

## 3. Results

### 3.1. Numerical Modal Analysis (FEA)

**Figure 9:**
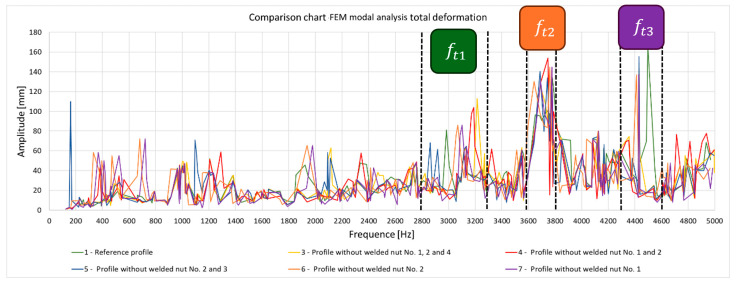
Overview of the maximum deformation of the system for all samples, showing the natural frequency for the individual samples in the f∈0−5000 Hz domain.

**Figure 10:**
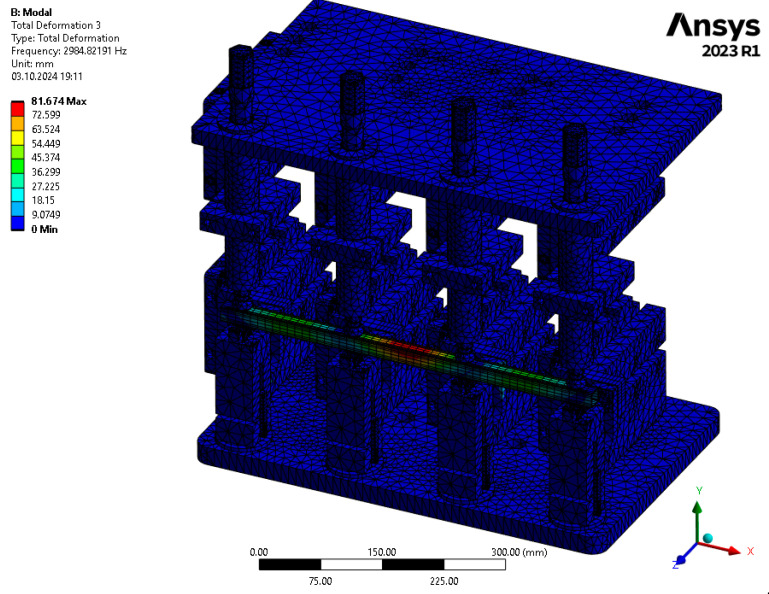
Total deformation for the form of vibration corresponding to the resonance frequency $f_{t1}=2894\;Hz$.

**Figure 11:**
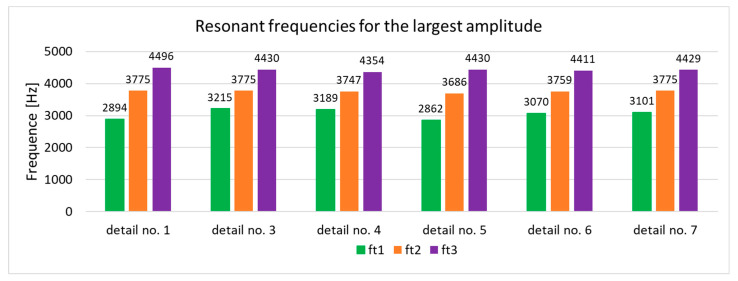
Summary plot of resonant frequencies for the largest amplitudes of total deformation.

The results of the numerical modal analysis provided 221 forms of vibrations for the model in the range of f∈0−5000 Hz. From the overview (Figure 9) of the total deformation, it is possible to see the frequency ranges for which quality control is possible using a suitable algorithm for signal analysis. Three intervals with a clear maximum of amplitude were noted: $f_{t1}\in 2800-3300\;Hz,\;f_{t2}\in 3600-3800\;Hz$, and $f_{t3}\;\in 4300-4600\;Hz$. The forms of vibrations for these resonance frequency intervals showed that only the range $f_{t1}\in 2800-3300\;Hz$ can be further investigated due to the fact that resonance exists throughout the test object. This form of vibration (Figure 10) allows an unambiguous determination of the mechanical properties of the welds as a result of a clear shift in the resonance frequency, depending on the connection between the profile and the nuts. A summary of the resonant frequencies of the individual details is shown in the diagram (Figure 11). It shows the exact values of the resonance frequency for the maximum displacement in the selected three intervals in order to highlight the differences that occurred as a result of incorrect connections between the samples. The absence of a waveform for sample No. 2 is due to numerical problems of a discontinuous system.

**Figure 12:**
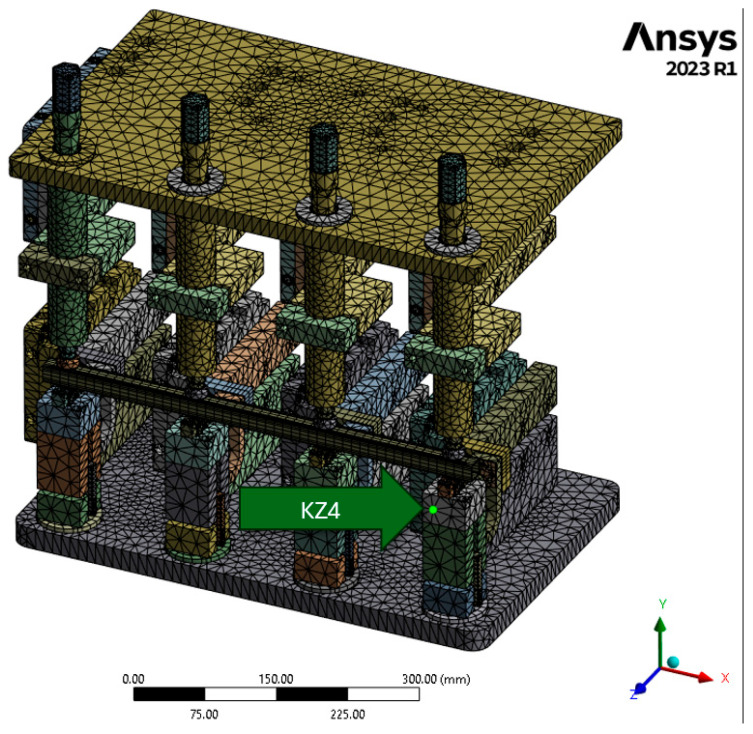
Depiction of the measuring point for deformation on the Z-axis of point KZ4.

**Figure 13:**
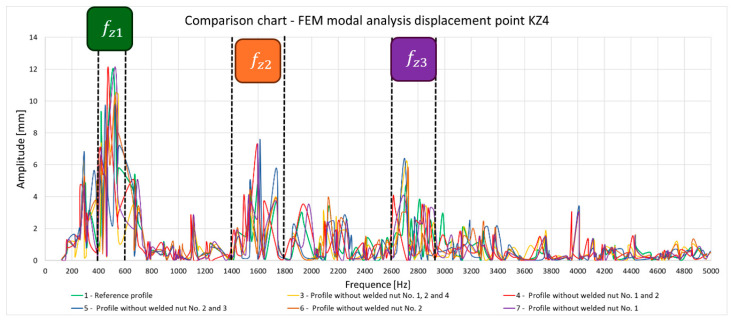
Overview of the maximum deformation of the system for all samples, showing the natural frequency for the individual figures in the f∈0−5000 Hz domain.

Subsequently, deformations were verified for point KZ4 (Figure 12), and the results are summarized in Figure 13.

**Figure 14:**
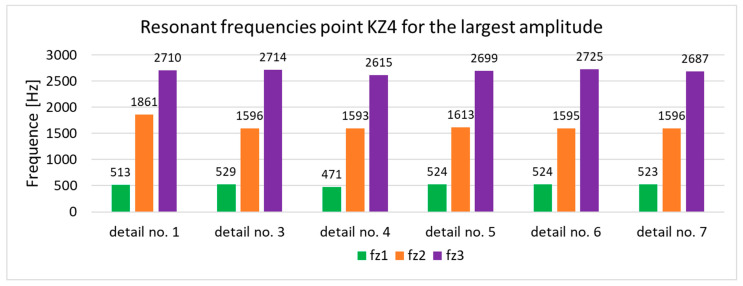
Summary plot of resonant frequencies for the largest amplitudes of total deformation.

All samples collected in the plot (Figure 13) show evidence of a change in resonant frequency due to a change in contact between the profile and the nut (Figure 14). If we take a closer look at the graph, we can distinguish three frequency ranges, $f_{z1}\in 400-600\;Hz$, $f_{z2}\in 1400-1800\;Hz$, and $f_{z3}\;\in 2600-2900\;Hz$, which additionally provide information about the location where the nut was missing. By analogy with the deformation of the entire test system, the deformation of point K_Z4_ for the form of vibration causing resonance on the welded workpiece is in the range of $f_{z3}\;\in 2600-2900\;Hz$, thus proving that the natural frequencies that are of interest for the assessment of workpiece quality are contained in the extended interval f ∈ (2400−3300 Hz).

**Figure 15:**
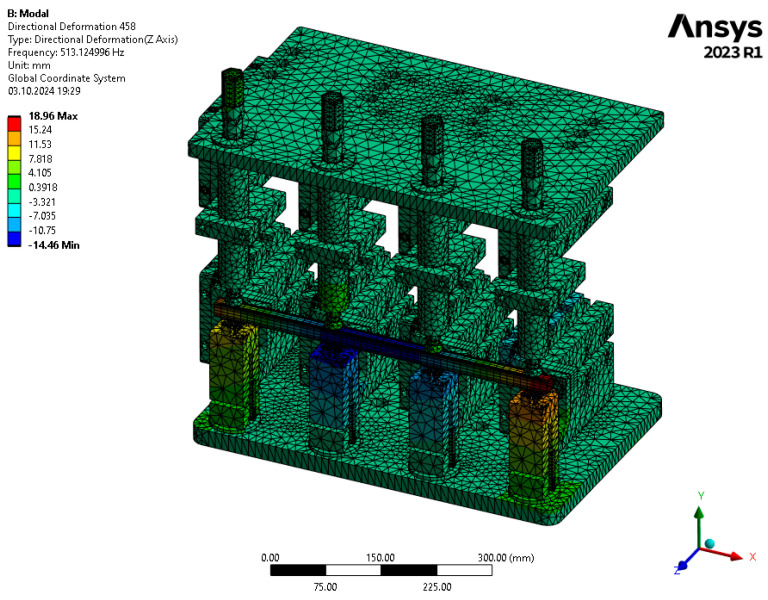
Directional deformation on the Z-axis for the form of vibration corresponding to the resonance frequency $f_{z1}=513\;Hz$.

In addition, modal analysis presents the possibility of assessing not only the forms that have the largest amplitude but also those that cause deformation on the test piece despite not having a large total displacement. The vibration form (Figure 15) for $f_{z1}\in 400-600\;Hz$ presents results that allow the quality of the component to be correctly assessed, particularly for the Z-axis. The problem of the fourth form is the lack of large displacements, which are clearly visible in the diagram showing total deformation.

### 3.2. Dynamic Resistance

**Figure 16:**
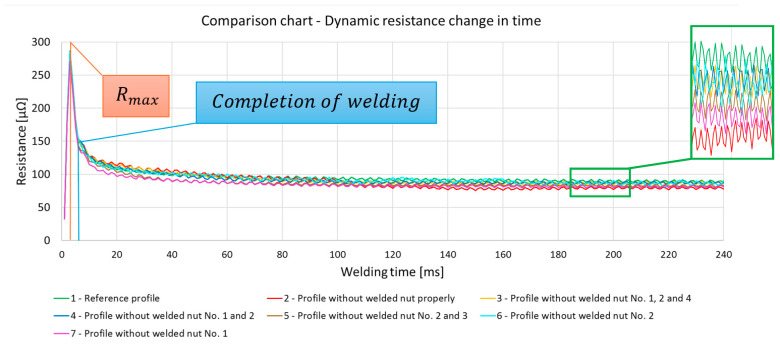
The dynamic resistance change over time for all details.

**Figure 17:**
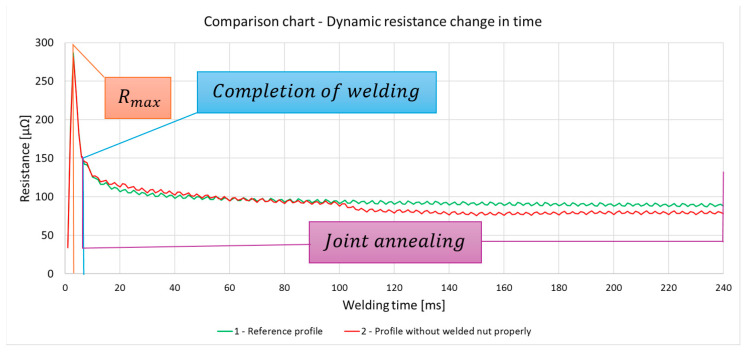
The dynamic resistance change over time for detail Nos. 1 and 2.

The analysis of the resistance change waveform, shown in the graph (Figure 16), allows observing that the process of joint formation takes 6 ms. During this time, the humps flow and settle, which is the result of the flow of electrical energy. At this point, the resistance reaches a peak value, followed by a decrease, leading to the maintenance of a constant resistance (within a certain tolerance range), which indicates the completion of the metallic joint formation process. The maintenance of a constant level of resistance after the completion of the joint formation indicates the annealing of the joint through the further flow of electrical energy, leading to the normalization of the joints (Figure 17).

**Figure 18:**
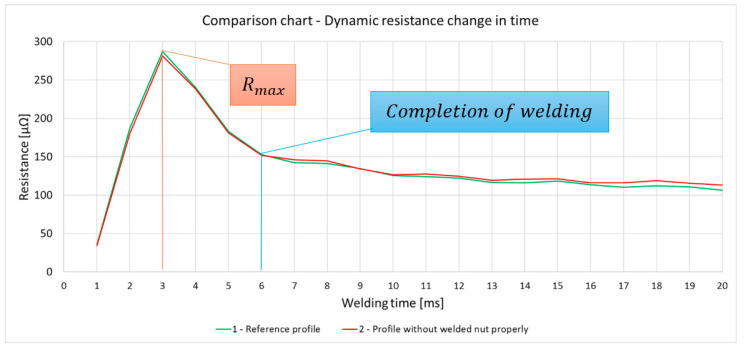
The dynamic resistance change over 20 ms for detail Nos. 1 and 2.

Analysis of resistance change over time for detail Nos. 1 and 2. (Figure 17) showed no statistically significant differences in maximum resistance between the analyzed elements. The comparison of element No. 1, which was the standard, with element No. 2, which showed a defect in all nuts (Figure 17), revealed that the difference in resistance is within the measurement error of 2%, which precludes the possibility of unequivocally identifying the quality of joints made by the multipoint-garb method on the basis of maximum resistance or the entire course of resistance change over time. Furthermore, the resistance changes waveform only in the time range in which weld formation occurs (see Figure 18). On this waveform, any characteristic differences are observed to evaluate the quality of the connections and the welding process.

**Figure 19:**
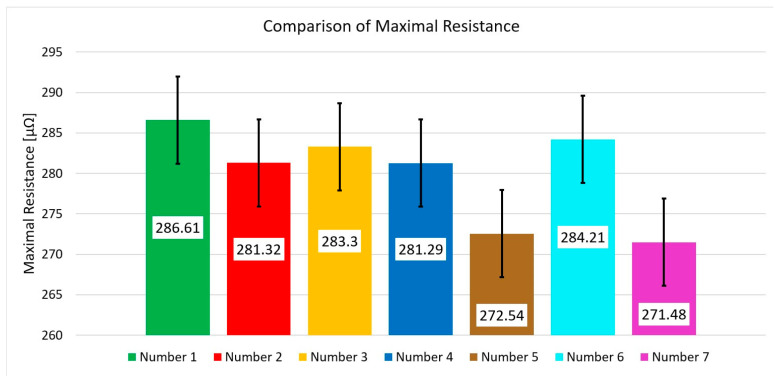
The comparison of maximum resistance values for all individual samples.

The comparison of maximum resistance (Figure 19) for the selected experimental samples shows a significant decrease in the maximum resistance value only for the elements Nos. 5 and 7. Nevertheless, the detailed characterization of the resistance waveform of these samples compared to the reference sample does not show any clear features that would allow an accurate assessment of the quality of the connection or indicate the presence of anomalies in the connectors.

The analysis of the results led to the following main conclusions:

- The technique of monitoring changes in resistance over time used to assess the quality of multi-point projection welded components does not allow an unambiguous assessment of the strength characteristics of a given component.
- The dynamics and short duration of the hump welding process, and thus the resistance changes over time, make it impossible to analyze the correctness of the process.
- Components with filed humps in the nut do not show a decrease or increase in maximum resistance during the welding process, which would allow an unambiguous identification of the occurrence of a defect during the process.
- The information obtained from the dynamic changes in resistance during the process does not provide data to identify in which of the four joints problems occurred during the welding process.

### 3.3. Experimental Modal Analysis

**Figure 20:**
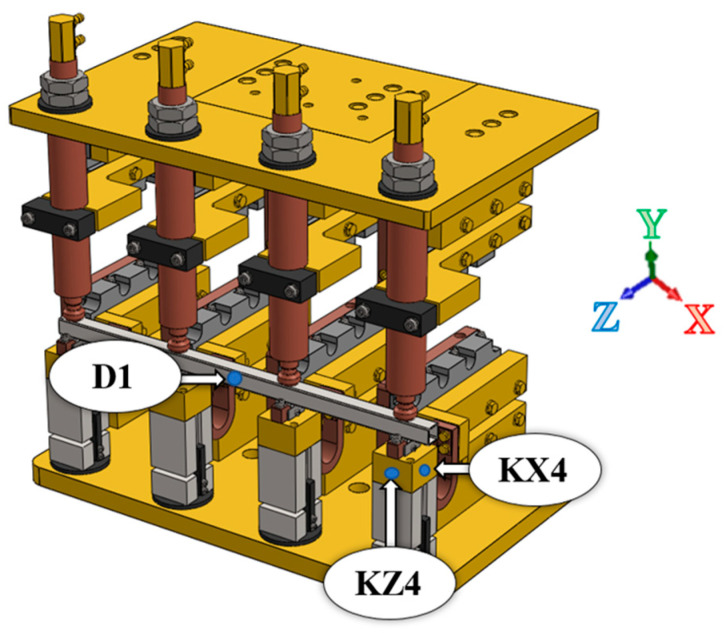
Diagram of the attachment of the accelerometer at point D1 and the points at which the KZ4 and KX4 systems are forced.

The modal analysis test took place after the welding process, with the resistance time history recorded. The accelerometer was mounted on the test piece at point D1, while the forcing was performed at points KZ4 and KX4 (Figure 20). Table 5 shows the waveforms with the resonance frequency of the individual samples and the superimposed white signal of the reference sample.

**Figure 21:**
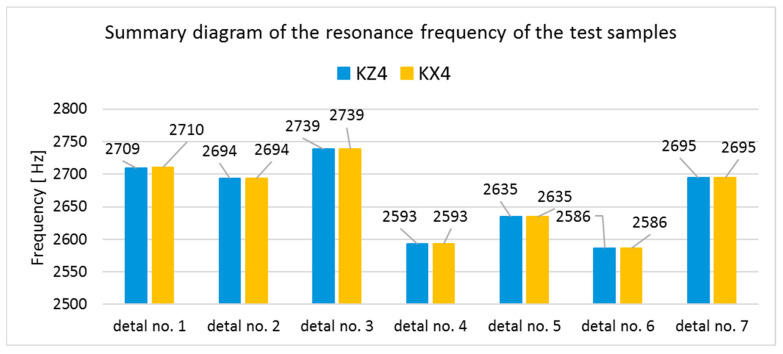
Diagram compiling the resonance frequencies of the test samples.

As a result of the inconsistencies in the tested samples, the modal analysis showed an overshoot of the resonance frequencies. A graph comparing the resonance frequencies of all tested components for two forcing points is presented in Figure 21. In addition to the differences in resonant frequency displacement, the individual samples show changes in the response waveform, which, when subjected to additional analysis, can provide additional information about the resulting connection. The response diagrams for point KZ4 and KX4 demonstrate the displacement of the resonance frequency as a result of the inconsistency in the profile-nut connection. This is particularly evident in the graph compiling the individual resonance frequencies (Figure 21). The resonance frequencies coincide with the interval selected in the tests using numerical modal analysis. All diagrams collected in Table 5 illustrate an additional vibration form for the interval $f_{1}\;\in \;(200-600\;Hz)$, which possesses a smaller amplitude, relative to the form appearing for the frequency $f_{1}\in \;(2400-3000\;Hz)$. The form for the $f_{1}$ interval, on preliminary tests, was observable when the accelerometer was fixed on a column on the Z-axis and also allows the quality of the components to be verified.

## 4. Discussion

The presented results confirmed the change in the resonance frequency for individual details with defects and those with correct welds. Signals from multiple accelerometer mounting points could provide a larger number of resonant frequencies for a given sample, which could enable future determination of the placement of non-welded joints.

Computer simulation allowed us to determine the mounting points of the accelerometer in order to obtain the best separation of spectra, enabling analysis of the quality of the detail after multi-spot welding.

The transparency of the frequency spectrum depends mainly on the design of the welding equipment. For this reason, the system and the workpiece had reduced stiffness, which reduced the mass contribution of the entire station to the system’s response, thus giving much clearer experimental results.

The technique of monitoring resistance changes over time used to assess the quality of multi-spot welded elements does not allow for a clear assessment of the quality of the element. The dynamics and short duration of the projection welding process, and therefore changes in resistance over time, make it impossible to assess the correctness of the multi-point process. Moreover, elements with filed humps in the nut do not show a decrease or increase in maximum resistance during the welding process, which would allow for clear identification of the occurrence of a defect during the process. The information obtained from dynamic resistance changes during the process does not provide data to identify which of the four joints experienced problems during the welding process.

Unlike resistance measurements, the spectral response of the system provides information about the distribution of the vibrating mass in space. In the future, this information can be used to control the position of joints, e.g., to check the position of the nut relative to the profile when the number of measurement locations in the system increases.

Moreover the frequency domain responses presented in the tests provide information such as local minima and the rate of rise of the slopes. These parameters are additional signal features that, when properly analyzed, can provide additional information about the connector in future studies.

## 5. Conclusions

Based on modern computer methods (FEM), a modelling of the effect of incompatibility on the resonant frequencies of an object in the form of a welding jig together with the workpiece can be carried out.

The results of the modal analysis carried out numerically are in agreement with the experimental results. The search intervals of the resonance frequencies that were selected in the numerical studies correctly estimated the range of values, as the resonance frequencies obtained from the experimental studies fell within it.

The tests carried out show that both the parallel to the accelerometer axis and the perpendicular direction provide a response in the frequency domain suitable for joint quality analysis. It makes sense to use multi-axis accelerometers in a future industrial application.

Comparing quality control by modal analysis against dynamic resistance, it can be determined that previous research demonstrates the advantage of modal analysis through higher efficiency and more information provided on component quality.

The research proves the possible industrial application of the method, which has a simple implementation due to the fact that only an accelerometer with a suitable measuring resolution is required.

The research work yielded additional information provided post-process, including information about inconsistencies during the multi-point projection welding process. This information will serve as corrective parameters, allowing the technologist to change the parameters to improve the quality of the joint.

## 6. Patents

The research work was carried out under patent “PL234863B1 Pressure welding machine for simultaneous multiple-element or multi-point resistance pressure welding and the method provided for this purpose”, concerning the part related to the quality control of the multi-point hump welding process.

## Figures and Tables

**Table 1:** Welding parameters for testing detail.

Process Parameter	Value	Unit
$\mathrm{Current}\;\mathrm{upslope}\;\mathrm{time},\;t_{rt}$	1	ms
$\mathrm{Current},\;I_{2}$	42	kA
Current flow time, t	240	ms
Electrode pressure force, F	12	kN
Number of pulses, -	1	-

**Table 2:** Modal hammer measuring characteristics.

Sensitivity	Frequency Range	Measuring Range	Resonant Frequency	Weight
11.2 mV/N	9.5 kHz	440 N	>15 kHz	100 g

**Table 3:** Acceleration sensor measuring characteristics.

Sensitivity	Frequency Range	Measuring Range	Resonant Frequency	Weight
10 mV/g	18 kHz	±500 g	85 kHz	4.75 g

**Table 4:** Measuring characteristics of the card.

Port In	Sampling Frequency	Power Current
3	102.4 kS/s	2–20 mA

**Table 5:** Plots of the frequency domain response of workpieces subjected to quality control using the dynamic resistance method, white line - test of reference sample, red line - first test, blue line - second test, green line – third test.

No.	Frequency [Hz]	Response Spectrum Plot from KZ4 Forced (White Signal is Sample no. 1 for Compere)
1	$f_{1.2}=2710$	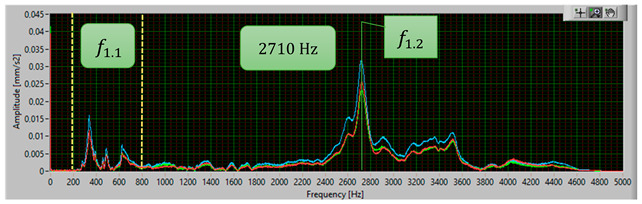
2	$f_{2.2}=2694$	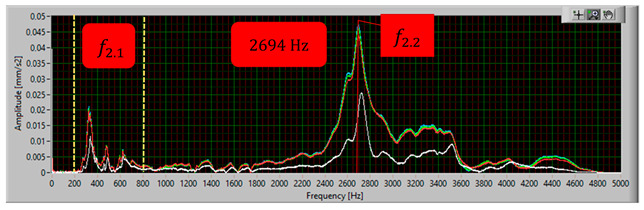
3	$f_{3.2}=2739$	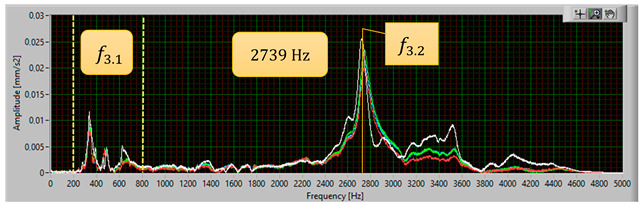
4	$f_{4.2}=2593$	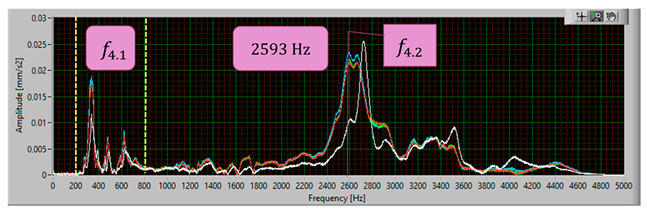
5	$f_{5.2}=2635$	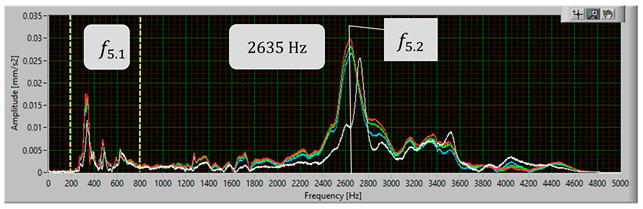
6	$f_{6.2}=2586$	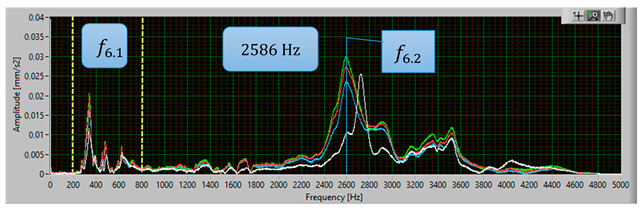
7	$f_{7.2}=2695$	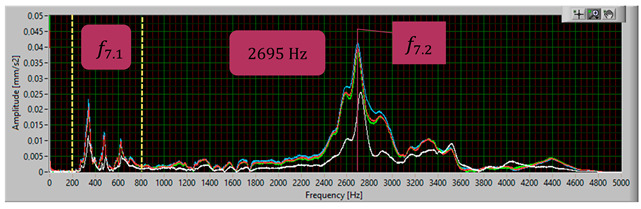

## Data Availability

The data generated and/or analyzed during the presented study are available from the corresponding author upon request.
